# The UK’s suitability for *Aedes albopictus* in current and future climates

**DOI:** 10.1098/rsif.2018.0761

**Published:** 2019-03-13

**Authors:** S. Metelmann, C. Caminade, A. E. Jones, J. M. Medlock, M. Baylis, A. P. Morse

**Affiliations:** 1 Institute for Infection and Global Health, University of Liverpool Liverpool, UK; 2 School of Environmental Sciences, University of Liverpool Liverpool, UK; 3 NIHR Health Protection Research Unit in Emerging and Zoonotic Infections, Liverpool, UK; 4 Medical Entomology Group, Public Health England, London UK

**Keywords:** *Aedes albopictus*, dynamic model, suitability analysis, diurnal temperature range, UK

## Abstract

The Asian tiger mosquito *Aedes albopictus* is able to transmit various pathogens to humans and animals and it has already caused minor outbreaks of dengue and chikungunya in southern Europe. Alarmingly, it is spreading northwards and its eggs have been found in the UK in 2016 and 2017. Climate-driven models can help to analyse whether this originally subtropical species could become established in northern Europe. But so far, these models have not considered the impact of the diurnal temperature range (DTR) experienced by mosquitoes in the field. Here, we describe a dynamical model for the life cycle of *Ae. albopictus*, taking into account the DTR, rainfall, photoperiod and human population density. We develop a new metric for habitat suitability and drive our model with different climate data sets to analyse the UK’s suitability for this species. For now, most of the UK seems to be rather unsuitable, except for some densely populated and high importation risk areas in southeast England. But this picture changes in the next 50 years: future scenarios suggest that *Ae. albopictus* could become established over almost all of England and Wales, indicating the need for continued mosquito surveillance.

## 1. Introduction

About 10 invasive species become established in Europe each year [1] and the UK alone spends about *£* 1.7 billion annually to mitigate their impacts [2]. One of these species that has already invaded Europe and might now spread to the UK is the Asian tiger mosquito, *Ae. albopictus*. This mosquito spreads worldwide through its long-lasting and drought-resistant eggs that can be transported over long distances, for example, in used vehicle tyres or lucky bamboo pot plants [3]. The eggs can also undergo a diapause to resist colder winter temperatures [4], allowing temperate regions significantly colder than its original niche in South East Asia to be colonized. In Europe, *Ae. albopictus* was introduced in the late 1970s to Albania [5], in 1990 to Italy [6] and more recently into greenhouses in the Netherlands [7]. Since its introduction into Italy, it has rapidly spread along the Mediterranean coast and is now expanding its northern range [8].

This is a major concern as *Ae. albopictus* is an effective disease vector. It can transmit a range of arboviruses affecting humans and animals, including chikungunya, dengue and Zika viruses [9], as well as filarial worms [10]. In Europe, it was responsible for two outbreaks of chikungunya in Italy and a few cases of dengue in Croatia and France in the last 10 years [11–13]. In addition, it is a potent vector of zoonotic diseases because it feeds on mammals, birds, reptiles and amphibians [14], although it feeds preferentially on humans in urban areas [15]. So whether or not *Ae. albopictus* will spread from continental Europe to the UK and subsequently become established is of significant public health interest. And there is evidence for recent introductions: in September 2016, eggs were found in Kent, the English county closest to France, by a surveillance team of Public Health England [16], followed by another finding of eggs and larvae in July 2017 at another site in the same county [17]. Here, gravid females have probably been carried over in cars or lorries and subsequently laid eggs when released at motorway service points.

Mechanistic and statistical niche models have been developed to analyse the UK’s climatic suitability for *Ae. albopictus*, suggesting that large parts of southern England are already suitable [18–20]. Dynamical models, better suited to capture the nonlinear behaviour of the mosquito’s development, have been published more recently [21–24]. While all of these models use seasonal or daily mean temperatures and rainfall as drivers, it has become clear that the diurnal temperature range (DTR) significantly affects the life cycle of insects too. The DTR is the difference between the maximum midday temperature and the minimum night-time temperature. Studies on *Aedes* mosquitoes show that rates for development and mortality differ substantially under constant temperature conditions compared with a realistic diurnal temperature cycle [25–27]. Models that already incorporate DTR have been developed for aphids [28], moths [29], generic insects [30] and its effect have been recently applied to a model for *Anopheles* mosquitoes [31].

Here, we describe the development of a novel dynamical model for *Ae. albopictus* that explicitly incorporates the effect of DTR on its life cycle. We use mosquito occurrence data and container index (CI) data to evaluate the model performance before analysing the suitability of the UK for this invasive mosquito under current climate conditions and under two climate projection scenarios for the future.

## 2. Model and methods

Based on previous studies, we chose a compartmental, climate-driven approach to model the life cycle of *Ae. albopictus* [21,23,24]. The model comprises five differential equations. Details on climate-dependent variables can be found in electronic supplementary material, SI.1.

### 2.1. Dynamic life cycle model

**Figure 1.**
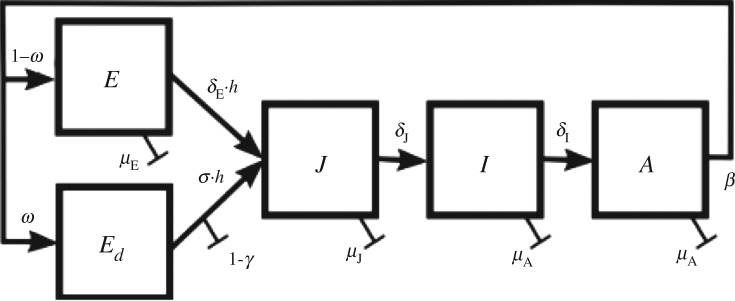
Life stages of *Ae. albopictus*. Eggs *E* hatch and become juveniles *J* (larvae and pupae). They develop to newly eclosed (immature) females *I* and finally to mature female adults *A*. Adult female mosquitoes lay normal eggs *E* in the summer months or diapausing eggs *E*_d_ at the end of the season. Diapausing eggs overwinter and are activated by a combination of longer day lengths, warmer temperatures and rainfall in spring.

The mosquito life cycle is described by five mosquito classes: normal, non-diapausing eggs *E*, juvenile aquatic stages *J*, immature female adults *I*, mature female adults *A* and diapausing eggs *E*_d_ (figure 1). Normal, non-diapausing eggs are laid during summer by mature females. Larvae hatch after eggs complete a development period and are activated by rainfall. The four larval stages and the pupal stage are combined into a single aquatic juvenile class in the model. Assuming a sex ratio of 50:50, juveniles then develop into newly eclosed male and female adults. Newly eclosed female mosquitoes do not directly show host-seeking behaviour. Instead, they first spend some time in a resting stage, only after which they take their first blood meal and start to lay eggs [32].

At the end of the season, the egg laying process depends on the photoperiod, *P*. When days are getting shorter, females start to lay diapausing eggs that do not hatch after a few days but overwinter. During the following spring, these eggs are ready to hatch when temperatures and photoperiod reach critical thresholds, and are eventually activated by rainfall.

All transitions from one class to another depend on temperature, *T*, and so do mortality rates. Because *Ae. albopictus’* water filled breeding sites are usually small [33], we use air temperature as a proxy for water temperature.

With parameter definitions given in table 1, model equations are as follows:

$$\begin{array}{rl} & \frac{\text{d}}{\text{d}t}E(t)=\beta (1-\omega )A(t)-h\delta_{E}E(t)-\mu_{E}E(t),\\ & \frac{\text{d}}{\text{d}t}J(t)=h\delta_{E}E(t)+h\sigma \gamma E_{d}(t)-\delta_{J}J(t)-\mu_{J}J(t)-\frac{J{\left(t\right)}^{2}}{K},\\ & \frac{\text{d}}{\text{d}t}I(t)=\frac{1}{2}\delta_{J}J(t)-\delta_{I}I(t)-\mu_{A}I(t),\\ & \frac{\text{d}}{\text{d}t}A(t)=\delta_{I}I(t)-\mu_{A}A(t)\\ \mathrm{and}\; & \frac{\text{d}}{\text{d}t}E_{d}(t)=\beta \omega A(t)-h\sigma E_{d}(t). \end{array}$$

**Table 1.** Parameter definitions and values. Derivation and references of parameters are shown in electronic supplementary material, SI.1. Environmental drivers are temperature, *T*, rainfall, *R*, photoperiod, *P*, latitude, *L* and human population density, *H*. Please note that the environmental carrying capacity, *K*, and the egg activation by rainfall, *h*, are defined in equations (2.1) and (2.2) further down in the manuscript.

	parameter	value/formula
CTT_S_	critical temperature over one week in spring (°C )	11.0^⋆^
CPP_S_	critical photoperiod in spring (hours)	11.25^⋆^
*σ*(*T*, *P*)	spring hatching rate (1/day)	$\{\begin{array}{ll} 0\; & \text{if }\;T_{7}<\mathrm{CT}T_{S}\;\text{or }\;P<\mathrm{CP}P_{S}\;\\ r_{S}=0.1^{†}\; & \text{if }\;T_{7}\geq \mathrm{CT}T_{S}\;\text{and }\;P\geq \mathrm{CP}P_{S}\; \end{array}$
CPP_A_(*L*)	critical photoperiod in autumn (hours)	10.058 + 0.08965 *L*
*ω*(*P*)	fraction of eggs going into diapause	$\{\begin{array}{ll} 0\; & \text{if }\;P>\;{\text{CPP}}_{A}\;\text{or }\;t<183\;\\ r_{A}=0.5\; & \text{if }\;P\leq \;{\text{CPP}}_{A}\;\text{and }\;t\geq 183\; \end{array}$
*δ*_E_	normal egg development rate (1/day)	1/7.1
*δ*_J_(*T*)	juvenile development rate (1/day)	1/(83.85 − 4.89 *T* + 0.08 *T*^2^)
*δ*_I_(*T*)	first pre-blood meal rate (1/day)	1/(50.1 − 3.574 *T* + 0.069 *T*^2^)
*μ*_E_(*T*)	egg mortality rate (1/day)	$-\ln(0.955\exp(-0.5\;{\left(\frac{T-18.8}{21.53}\right)}^{6}))$
*μ*_J_(*T*)	juvenile mortality rate (1/day)	$-\ln(0.977\exp(-0.5{\left(\frac{T-21.8}{16.6}\right)}^{6}))$
*μ*_A_(*T*_mean_)	adult mortality rate (1/day)	$-\ln(0.677\exp(-0.5{\left(\frac{T_{\mathrm{mean}}-20.9}{13.2}\right)}^{6})\;T_{\mathrm{mean}}^{0.1})$
*γ*(*T*_DJF,min_)	survival probability of diapausing eggs (1/winter)	$0.93\exp(-0.5{\left(\frac{T_{\mathrm{DJF},\min}-11.68}{15.67}\right)}^{6})$
*β*(*T*)	egg laying rate (1/day)	$\{\begin{array}{ll} 33.2\;\exp\left(-0.5{\left(\frac{T-70.3}{14.1}\right)}^{2}\right){\left(38.8-T\right)}^{1.5}\; & \text{if }\;T\leq 38.8\;\\ 0\; & \text{if }\;T>38.8\; \end{array}$
*λ*	capacity parameter (larvae · days /hectare)	10^6^${\;}^{\text{‡}}$

^⋆^[34].

${\;}^{†}$Best estimate.

${\;}^{\text{‡}}$[22,35].

Development rates, *δ*, and mortality rates for eggs and juveniles, *μ*_E_ and *μ*_J_, depend on the actual oscillating diurnal temperature *T*. The development from juvenile to immature females is halved in the equation for (d/d*t*)*I*(*t*), (1/2)*δ*_*J*_, to account for the 50:50 sex ratio. Only the mortality rate for adults is derived from field data that already include a DTR. Daily mean temperatures, *T*_mean_, are, therefore, used for *μ*_A_. *T*_7_ is the average temperature over the recent 7 days, used to trigger the spring hatching rate.

Owing to the lack of information regarding the survival rates of eggs over long time periods (several months), we assume a survival probability *γ* of diapausing eggs that is dependent on the minimum winter temperature experienced, *T*_DJF,min_. The survival probability is applied when eggs are activated in spring, see electronic supplementary material, SI.1 for details. Remaining diapausing eggs that have not hatched until August are removed.

Larval mortality not only depends on temperature but also on an environmental carrying capacity, *K*, representing juvenile competition and predation [36]. We use the model by White *et al.* [37] and its extension by Erguler *et al.* [24] to calculate *K* from rainfall, *R*, and human population density, *H*

2.1 $$K(R,H)=\lambda \frac{1-\alpha_{\mathrm{evap}}}{1-\alpha_{\mathrm{evap}}^{t}}\sum_{x=1}^{t}\alpha_{\mathrm{evap}}^{\left(t-x\right)}(\alpha_{\mathrm{rain}}R(x)+\alpha_{\mathrm{dens}}H(x)).$$

As we model mosquito abundance in individuals per hectare, we keep the parameters at *α*_evap_ = 0.9, *α*_dens_ = 0.001 km^2^ and *α*_rain_ = 0.00001 mm^−1^ [24] but multiply by a scaling factor *λ* to reach a maximum carrying capacity ranging between 500 000 and 800 000 individuals per hectare [22,35].

Similar to the carrying capacity, we model the hatching of eggs depending on rainfall and human population density. We use the rainfall-dependent approach by Abdelrazec & Gumel [38] and assume that up to $\epsilon_{\mathrm{rat}}=20\text{\%}$ of eggs can hatch in densely populated areas regardless of rainfall conditions:

2.2 $$\begin{array}{rl} h(R,H) & =(1-\epsilon_{\mathrm{rat}})\frac{\left(1+\epsilon_{0})\exp(-\epsilon_{\mathrm{var}}{\left(R(t)-\epsilon_{\mathrm{opt}}\right)}^{2}\right)}{\exp(-\epsilon_{\mathrm{var}}\;{\left(R(t)-\epsilon_{\mathrm{opt}}\right)}^{2})+\epsilon_{0}}\\ & \;+\epsilon_{\mathrm{rat}}\frac{\epsilon_{\mathrm{dens}}}{\epsilon_{\mathrm{dens}}+\exp(-\epsilon_{\mathrm{fac}}H(t))}. \end{array}$$

We set the optimal amount of daily rainfall to *ε*_opt_ = 8 mm, and use *ε*_0_ = 1.5 and *ε*_var_ = 0.05 mm^−2^ [38]. Density-dependent parameters are set to *ε*_dens_ = 0.01 and *ε*_fac_ = 0.01 km^2^, such that egg hatching is increased in areas where *H* > 500 people per km^2^.

Note that other studies split the juvenile stage into larvae and pupae and some also split the mature female stage into host seeking, gestating, and ovipositing stages [22–24]. We also simulated these scenarios but they did not improve model fit to presence or CI data. As there was also more parameterization data available for a reduced model, we kept the model framework with a minimum number of equations. See electronic supplementary material, SI.2 for further details.

The model is implemented in *Octave v4.2.1* and Runge–Kutta 4 is used to solve ODEs. All scripts and a short example can be found in the electronic supplementary material.

#### 2.1.1. Suitability index

We propose a suitability index *E*_0_ that relates to the basic reproduction number *R*_0_ in epidemiological studies. In epidemiology, *R*_0_ is defined by the number of susceptibles infected by a single infectious individual in an otherwise uninfected population. Accordingly, we define our suitability index by the number of eggs that are produced at the end of a year, after placing a single (diapausing) egg at the beginning of the year into an uncolonized location. The amount by which the number of eggs has increased (suitable) or decreased (unsuitable) defines the suitability index *E*_*i*_ of that year *i*. Repeating this procedure for *n* consecutive years and taking the geometric mean of the yearly suitability indices gives the suitability index, *E*_0_, for the according period,

$$E_{0}=n\sqrt{\Pi_{i=1}^{n}E_{i}},$$

with *E*_*i*_ = *E*_d_(day = 365)/*E*_d_(day = 1). Note that the crucial scaling of *E*_0_ depends on the carrying capacity, *K*. With our standard settings, the model predicts about 1200 adult female *Ae. albopictus* per hectare for August/September in Rome (figure 7). This is well in the range of mark–release–recapture data, with an estimated 1400 females per hectare [39]. See electronic supplementary material, SI.3 for further details.

#### 2.1.2. Diurnal temperature cycle

To calculate the DTR, we use the model by DeWit [40], which is well suited to compute realistic temperatures throughout the day from maximum and minimum temperatures [41]. Time points for temperature calculation are chosen according to the time steps for our explicit numerical solver, e.g. if $k=\frac{1}{100}$, we calculate 100 actual temperatures throughout the day at 0.14, 0.19, … 24.00. Temperatures during day *i* are calculated by

$$T_{i}(h_{t})=\left\{ \begin{array}{ll} \frac{T_{i-1}^{\max}+T_{i}^{\min}}{2}+\frac{T_{i-1}^{\max}-T_{i}^{\min}}{2}\cos\left(\frac{h_{t}\;+\;10}{10\;+\;t_{s}}\pi \right) & \text{if}\;h_{t}<t_{s}\\ \frac{T_{i}^{\max}+T_{i}^{\min}}{2}-\frac{T_{i}^{\max}-T_{i}^{\min}}{2}\cos\left(\frac{h_{t}\;-\;t_{s}}{14\;-\;t_{s}}\pi \right) & \text{if}\;t_{s}<h_{t}<14\\ \frac{T_{i}^{\max}+T_{i+1}^{\min}}{2}+\frac{T_{i}^{\max}-T_{i+1}^{\min}}{2}\cos\left(\frac{h_{t}\;-\;14}{10\;+\;t_{s}}\pi \right) & \mathrm{else} \end{array}\right.$$

with $T_{i}^{\max/\min}$ being the maximum or minimum temperature of day *i*. The model assumes *T*^min^ at sunrise *t*_s_ and *T*^max^ at 14.00 local time. The time of day in hours is given by *h*_t_, and the time of sunrise, *t*_s_, is calculated using the daylight model by Forsythe *et al.* [42], depending on latitude, *L*, and the day of year, DOY. See electronic supplementary material, SI.4 for further details on the daylight model equations.

#### 2.1.3. Climate and population density data

We run our model with a range of different climate data sets from historical records and future climate projections. For mosquito suitability in the UK, we compare the observed gridded climate datasets from E-OBS on a 25 × 25 km spatial scale [43] and from UKCP09 on a 5 × 5 km scale [44]. The E-OBS dataset is used for model validation over Europe and the ERG5 Eraclito dataset [45] is used for the model runs in the Emilia-Romagna region.

For future model runs across Europe, we use 25 × 25 km spatial scale climate projections from the NASA NEX-GDDP project [46] for two different emission scenarios, the medium RCP4.5 and the extreme RCP8.5 scenario. A subset of five general circulation models from the full set of 21 was chosen to represent the full range of uncertainty, see electronic supplementary material, SI.5 for details. For future changes, we focus on the period 2060–2069, the 2060s hereafter.

Human population density is based on the GPWv4 dataset [47]. For the 2060s projections, we assume the total UK population has increased from 65.5 million to 75 million [48] but has not changed in its spatial distribution.

### 2.2. Validation

#### 2.2.1. Mosquito data

To validate the spatial distribution of suitability simulated by the model, we used *Ae. albopictus* occurrences [49], updated with data from the recent literature [16,17,50–53], and classified into established populations and one-time sightings according to the 2018 ECDC classification [8]. Occurrence points that were less than 25 km apart from one another were clustered together, resulting in a total of 234 out of 385 data points. We then checked whether each established occurrence point fell into a grid cell that was calculated to be suitable (*E*_0_ > 1).

**Figure 2.**
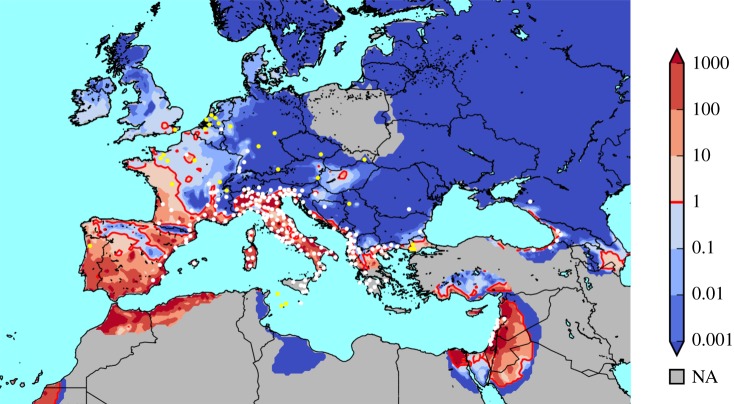
Spatial validation. White dots show established *Ae. albopictus* populations, yellow dots show one-time sightings. Background colours show the simulated suitability index of the period 2006–2016. Red contour distinguishes suitable (*E*_0_ > 1) from unsuitable areas (*E*_0_ < 1). In the grey area, climate data from the E-OBS dataset was incomplete for all years of the study period. (Online version in colour.)

Figure 2 shows the suitability index for the period 2006–2016, which is highly consistent with occurrence data: 83% of the established populations fall into a suitable grid cell, 17% into unsuitable ones (excluding grid cells that are not covered by climate data). However, the model misses some points in the southern Alps, the Bulgarian/Romanian Black Sea coast and some southern German cities. This is possibly because occurrences fall into warmer valleys or urban areas with microclimate conditions that are not captured by the coarse spatial resolution of the climate data. Also, the model predicts suitable conditions for areas such as southern Germany in most years but specific years with a very cold winter or dry summer lower the 10-year suitability index (compare electronic supplementary material, figure S7).

More densely populated areas, such as Madrid, Paris and London appear as suitable; they act as heat islands, further increasing mosquito development [54], and they supply mosquito breeding sites by man-made containers and irrigation.

We used observed CI data that are available for northern Italy to validate our model not only in space but in time (see electronic supplementary material, SI.7). While the onset and end of the mosquito season is well captured by the model, it sometimes over- or underestimates the peak in mosquito numbers at interannual timescale. The Pearson correlation between observed and simulated egg data is *r* = 0.70 (95% CI: 0.67 ≤ *r* ≤ 0.73, *N* = 996).

#### 2.2.2. Sensitivity analysis

To investigate the influence of each parameter on the final model output, *E*_0_, we perform the elementary effects test (EET) [55]. The EET measures the influence of single input parameters on model outputs, as well as their degree of interaction with other parameters. Latin hypercube sampling is used to vary parameters in the range of ±10% of the standard setting [56]. The model is then run with the Italian climate data until convergence and the total egg number after 5 years is taken as reference. *Octave* scripts for these methods come from the SAFE toolbox [57].

**Figure 3.**
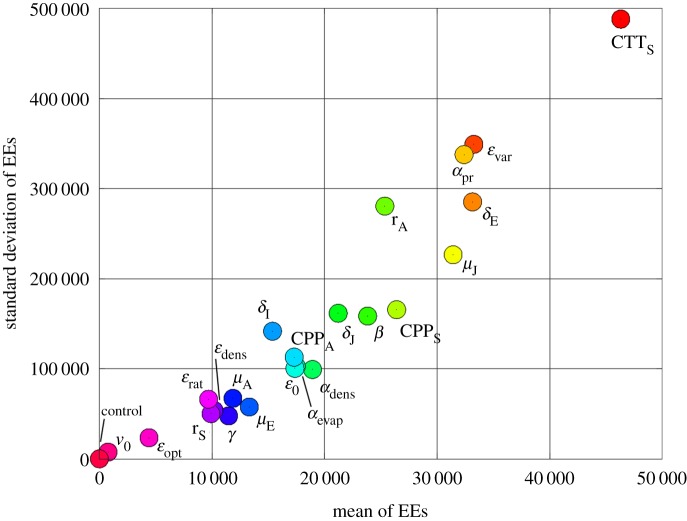
Elementary effects test. The higher the mean EEs, the more influential the parameter on the model outcome, *E*_0_. The higher the standard deviation of the EEs, the larger its degree of interactions with other parameters. (Online version in colour.)

The critical temperature threshold in spring, CTT_S_, has the biggest effect on *E*_0_, followed by parameters determining rainfall dependencies such as *ε*_var_ and *α*_rain_, and egg development, *δ*_E_ (figure 3). Other mosquito-specific parameters range in the middle. Parameters such as initial egg numbers, *v*_0_, or other hatching rate parameters, *ε*_dens_, *ε*_rat_ and *ε*_opt_, have a limited impact on the model output for the Italian climate settings. The distributions for mean and standard deviation of EEs indicate that parameters with a bigger effect on other parameters have a bigger effect on the model output, *E*_0_.

## 3. Results

### 3.1. Diurnal temperature range

To analyse the effect of the DTR on mosquito population size, we first run the model under constant conditions (5 mm rainfall per day, 12 h daylight, 100 humans per km^2^, starting with 1 egg per hectare) for a range of different temperatures. The model is run with constant mean temperatures (DTR = 0°C) and afterwards with oscillating temperatures (0°C < DTR ≤ 12°C), simulating the diurnal temperature cycle. We then compare absolute mosquito numbers after 365 days by dividing egg numbers that experienced DTR by egg numbers at constant temperatures.

**Figure 4.**
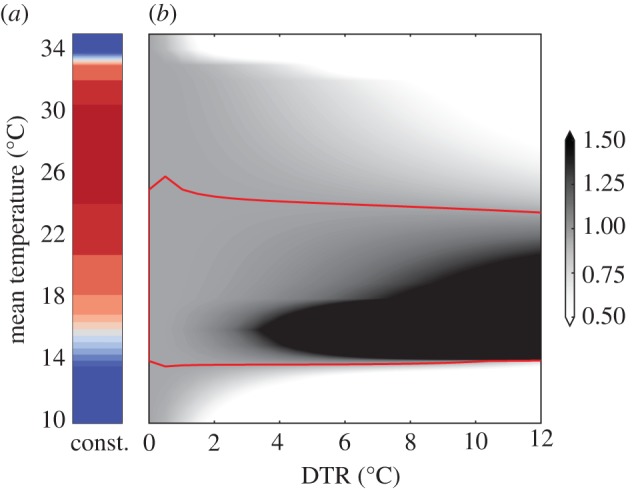
DTR impact on mosquito numbers. (*a*) Population size of *Ae. albopictus* measured in *E*_0_ at constant temperature, with colour coding as in figure 2. (*b*) Relative population size after 365 days with diurnal temperature cycle compared to the population size experiencing constant temperatures (*a*). Values above 1 (within the red contour line) indicate where oscillating temperatures increase the population size. Mean temperature is given on the *y*-axis and the DTR is given on the *x*-axis. (Online version in colour.)

Figure 4 shows that oscillating temperatures have a positive effect on the population size at lower mean temperatures, roughly for 14°C < *T*_mean_ < 24°C. This is actually the lower bound of the mosquito’s suitable temperature niche, equilibria and stability analyses show that mosquito populations could survive at constant temperatures between approx. 13°C and 32°C (see electronic supplementary material, SI.9). Only when temperatures are very low (*T* < 13°C), DTR has a negative effect on the population numbers as mosquitoes experience high mortalities at the reached minimum temperatures. Electronic supplementary material, figure S12 shows more detailed time series of population growth at different temperature scenarios, these time series have been used to create figure 4. Including the DTR in simulations increases the suitability especially in northern regions compared with model runs that only use daily mean temperatures (electronic supplementary material, figure S11).

### 3.2. Current suitability of the UK

**Figure 5.**
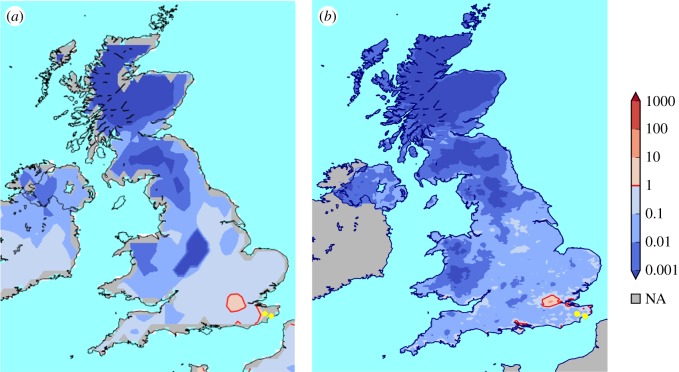
Suitability of the UK. Comparison of UK mosquito suitability at different spatial resolutions for the years 2006 until 2016, using E-OBS (*a*) and UKCP09 (*b*) climate data. Yellow dots show locations where *Ae. albopictus* has been found in 2016 and 2017. (Online version in colour.)

To analyse the UK’s suitability for this mosquito, we run our model with two climate datasets for the recent period 2006–2016. Figure 5 shows that simulations driven by climate datasets with high and low spatial resolution agree in that the London area, the Thames estuary and parts of the southern coast are already suitable for the mosquito. Other warmer areas around the Severn estuary or in East Anglia, as well as populated northern regions such as Merseyside or around Sheffield are close to but not yet suitable. The Scottish Highlands, the Pennines and the Welsh mountains are unsuitable. Note that we are looking at a 10-year period to analyse the suitability for long-term establishment. We can also look at individual years, finding, for example, that 2016 was suitable over a larger region of the UK (see electronic supplementary material, figure S7).

### 3.3. Future suitability of the UK

**Figure 6.**
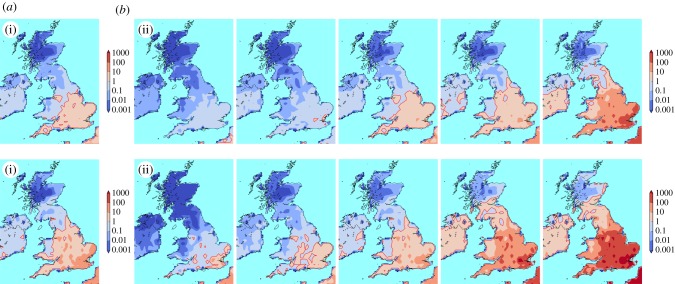
Future suitability of the UK. Suitability index for 2060–2069. (*a*) Geometric mean over all five model outputs for RCP4.5 (i) and RCP8.5 (ii). (*b*) Suitability index shown for each climate model individually for RCP4.5 (i) and RCP8.5 (ii). Left to right: minimum, 25th quantile, median, 75th quantile, maximum temperature increase for the British Isles. Climate models in order from the coldest to warmest are inmcm4, MRI-CGCM3, NorESM1-M, CanESM2, MIROC-ESM-CHEM for RCP4.5, and inmcm4, CESM1-BGC, NorESM1-M, CanESM2, MIROC-ESM-CHEM for RCP8.5. (Online version in colour.)

Figure 6 shows the UK’s future mosquito suitability for two emission scenarios, RCP4.5 and RCP8.5, for the 2060s.

Compared with recent UK suitability (figure 5), most of England will have become suitable for the establishment of *Ae. albopictus* populations in about 50 years when looking at the means. Parts of Wales might become suitable, depending on the emission scenario. Scotland and Northern Ireland remain mostly unaffected. However, there are large differences across the five climate models: only the southeast tip of the UK will become suitable with the coldest climate model, while almost the whole UK will become suitable with the warmest model.

**Figure 7.**
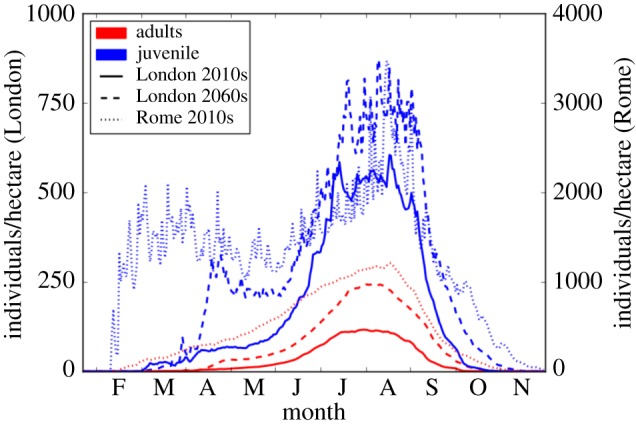
Mosquito season. Comparing the simulated length of the mosquito season in London in the 2010s and 2060s and with Rome in the 2010s. Means of 10 years for London and Rome 2010s data, based on E-OBS climate data. Future estimates are based on the ensemble mean of five RCP4.5 projection runs for 2060s. Note the different *y*-axis for London and Rome. (Online version in colour.)

Focusing on changes in seasonal abundance, simulations indicate that in current London, *Ae. albopictus* population sizes would be small in early summer and reach relative high number in July and August (figure 7). Future scenarios show an expansion of this peak into September and an overall increase in numbers. However, the length of the peak mosquito season would be short and population sizes remain low with respect to simulated values in Rome for recent climate conditions. Simulations for figure 7 were started 1 year ahead of the analysed period and mosquito numbers transferred from the end of a year into the next.

## 4. Discussion

Numerous studies investigating the climatic dependencies of *Ae. albopictus* have been published in recent years [4,58–63]. Taking these new findings into account and building on other modelling studies [21,23,24], we developed a dynamical model for *Ae. albopictus* that explicitly simulates the effects of rainfall for egg hatching and larval development, photoperiod for diapause induction and ending, and considers minimum and maximum temperatures that shape mortality and development rates of aquatic and adult stages.

The full temperature range experienced by mosquitoes in the field tend to increase model development rates throughout all stages. Mosquito populations at the lower temperature range (14°C to 24°C) develop better with oscillating temperatures. Here, night-time temperatures do not affect the development rates that are quite low anyway, while higher temperatures during the day significantly increase them [31]. Conversely, when mean temperatures are already high, lower night-time temperatures decrease development rates, while even higher temperatures during the day tend to increase mortality rather than development rates [27]. Thus, the DTR can be crucial for suitability analyses and should be considered for modelling the life cycle of mosquitoes and other insects [30,31], as it has already been done for the modelling of temperature-dependent viruses or malaria protozoans that mosquitoes can transmit [64–66].

Looking at the UK climate conditions for the past 10 years, we find large parts of the UK rather unsuitable for *Ae. albopictus*, except for some warmer and densely populated areas in the southeast of England. This finding suggests the mosquito has to be introduced into specific areas to enable long-term establishment. This result differs from findings by other modelling studies showing a medium to high suitability of larger parts of England [19,20,24,67] with up to five months adult mosquito activity in certain areas [18].

Our results are a bit more conservative because we included a rainfall-dependent mechanisms for egg hatching and larval mortalities in the model. Instead of constant egg hatching, we assumed that rainfall events lead to eggs being submerged under water and subsequent hatching. Similar to the finding of Tran *et al.* [22], the introduction of a rainfall-dependent egg hatching rate does not improve the model output fit to empirical abundance or ovitrap data. However, we found it enhances model performance in arid and unpopulated areas such as central Spain and Turkey.

We further assumed that a high human population density positively influences both the hatching of eggs and the survival of larvae because the mosquito is able to develop indoors [68], but also in arid but densely populated areas, where water storage and sprinkling create breeding habitats [69].

While large parts of England might not yet be suitable for a long-term establishment of this mosquito, individual years (especially the warmer recent ones, like 2016) already show a higher suitability which will continue to increase in the future [70]. Looking 50 years ahead, our projections suggest that *Ae. albopictus*, if introduced, could establish itself over most of England and southern Wales during the 2060s. The mosquito could become abundant in London during future summers; but even severe warming scenarios suggest that population sizes would still remain small with respect to current conditions in Rome, Italy. Large uncertainties related to the selected climate model and the emission scenario are due to the large variability of rainfall and temperature projections in the multi-model ensemble.

The question whether *Ae. albopictus* is able to spread from continental Europe to England is of great importance for public health and veterinary services. This mosquito is a vector that can transmit pathogens that are present or constantly introduced into the UK, such as several arboviruses like Zika, dengue and chikungunya [71] and the canine heartworm *Dirofilaria immitis* [72]. Moreover, it is a very competitive species that could replace endemic mosquito species and become a biting nuisance to the local population [73]. Finding parts of southeast England already suitable and predicting a strong increase in suitability for most of England in the future, we highly recommend stringent vector surveillance in southern UK ports and high importation risk areas along motorways [3,74]. In addition, human and veterinary health services should get prepared to deal with pathogens transmitted by *Ae. albopictus* in warm summers [75], as it is recently happening in southern European countries.

## Supplementary Material

Supplementary Information

## Supplementary Material

Ae. albopictus Model

## Data accessibility

The E-OBS climate dataset for Europe is publicly available, following registration, at https://www.ecad.eu/download/ensembles/ensembles.php. The UKCP09 climate dataset for the UK is available, following registration, at https://www.metoffice.gov.uk/climatechange/science/monitoring/ukcp09/download/index.html. The ERG5 Eraclito climate dataset for the Emilia-Romagna region is publicly available at https://www.arpae.it/dettaglio_documento.asp?id=6147&idlivello=1528. The climate projections of the NASA NEX-GDDP project are available, following registration, at https://cds.nccs.nasa.gov/nex-gddp/. Population density data from GPWv4 is publicly available, following registration, at http://sedac.ciesin.columbia.edu/data/collection/gpw-v4. Model scripts are available in the electronic supplementary material.
