# Supplementary material for: The UK’s suitability for Aedes albopictus in current and future climates
Source: J R Soc Interface. 2019 Mar 13;16(152):20180761. doi: 10.1098/rsif.2018.0761 (PMC6451397; doi:10.1098/rsif.2018.0761)
Supplement: Supplementary Information [file rsif20180761supp1.pdf]

Supplementary Information

The UK's suitability for *Aedes albopictus* in current and future climates

Journal of the Royal Society Interface

Metelmann, Caminade, Jones, Medlock, Baylis, Morse

SI.1 Model parametrisation

Development and fecundity rates

According to Quinn [72], development rates do not continuously increase with increasing temperature but show an optimum after which they decrease again. We thus choose a quadratic polynomial function $d(T) = a \cdot T^2 + b \cdot T + c$ for development periods as shown in Figure S1, and took the inverse to get development rates, $\delta(T) = 1/d(T)$. Data for egg development did not show a clear relationship with temperature, so we chose the median of all data points as a constant.

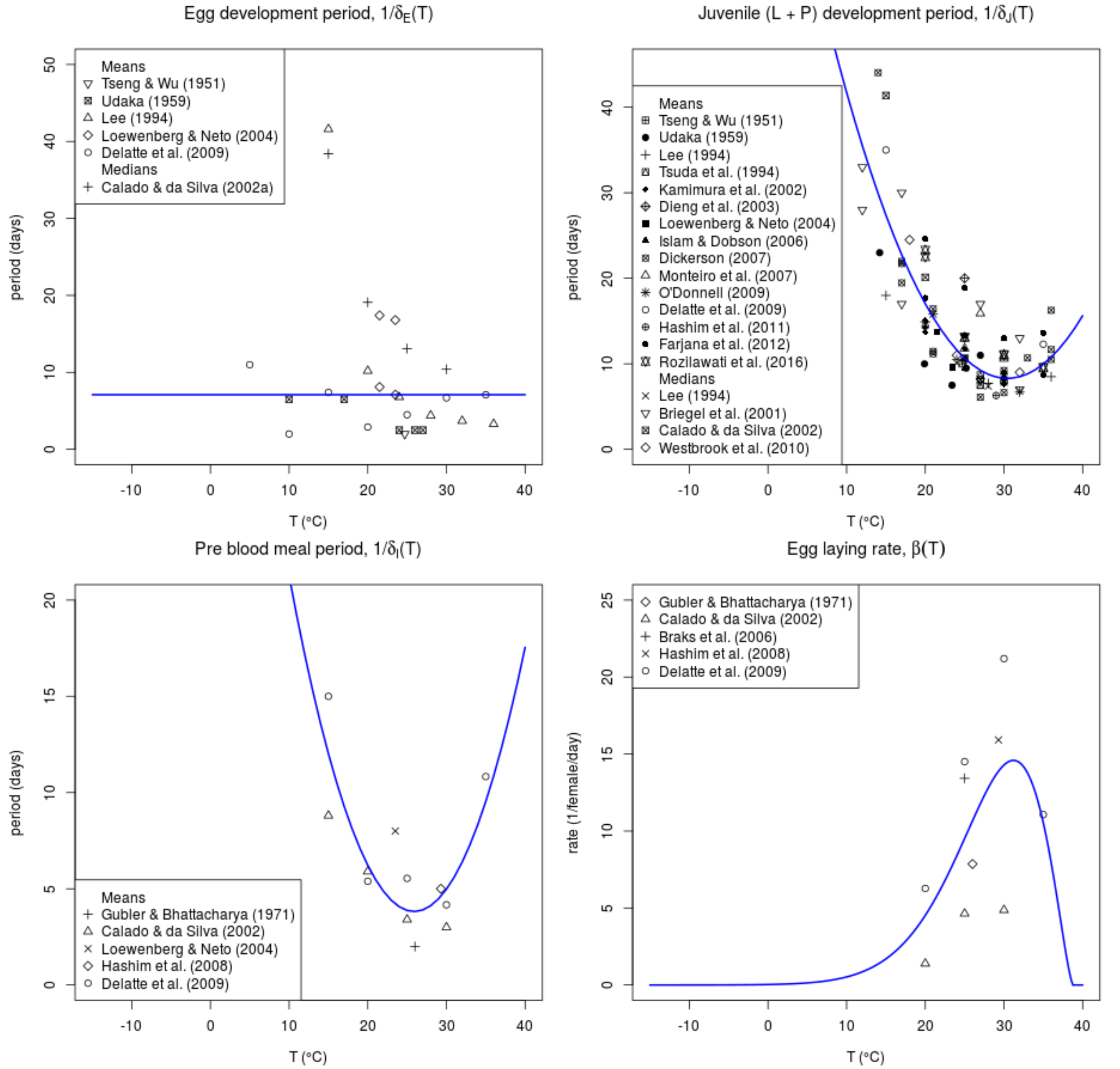

Figure S1: Development periods for *Ae. albopictus* eggs, juveniles, and immature females. Egg development did not show a clear correlation with temperature, so we chose a constant period. Bottom right: female fecundity given by eggs per mature female per day.

Egg laying rates were averaged over the whole lifetime of a mature female, excluding the first pre-bloodmeal

period. A skewed Gaussian distribution was fitted to the data.

Survival rates

Fitting functions to daily survival rates derived from laboratory data were of the form $s(T) = a \cdot \exp(-(\frac{T-b}{c})^6)$ (Fig. S2). We use a sextic rather than a quadratic exponent as data suggest an optimal plateau rather than a peak around an optimal temperature. The daily survival probabilities are subsequently translated into mortality rates suitable for a differential equations setting by taking the negative natural logarithm, $\mu(T) = -\ln(s(T))$.

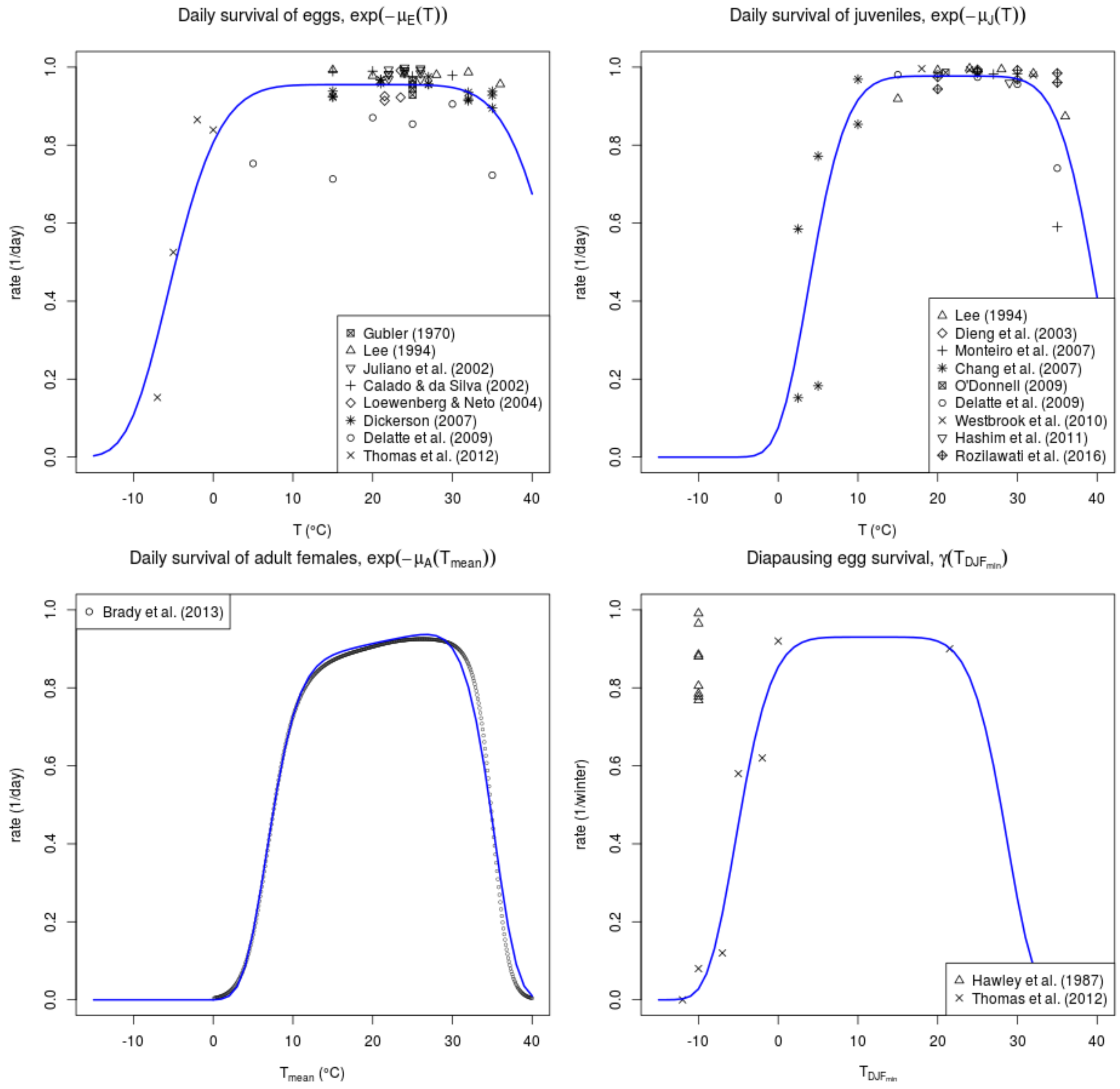

Figure S2: Daily survival probabilities for summer eggs, juveniles, and adults and winter survival probability for diapausing eggs. Data points from Chang *et al.* [95] are only for L1 and L4 larvae.

For daily survival of adult mosquitoes, we only use the meta analysis of Brady *et al.* [98] as they investigate survival in the field which we are interested in. The field survival rates are calculated for daily or even monthly mean temperatures [99, 100, 101, 102, 103], so we decided to use daily mean temperatures to calculate adult mortality rates, rather than the full diurnal temperature cycle. We used a temperature-dependent factor of $T^{0.1}$ to account for the skewness in the daily survival probability, $\mu_A(T) = -\ln(s_A(T) \cdot T^{0.1})$.
Finding a temperature-dependent curve for diapausing egg survival was the most challenging task as there are no laboratory studies investigating the survival at constant temperatures for periods of up to five months. However, some studies analyse the effect of sub-zero treatments on the eggs' survival rates [104, 105, 106]. They suggest that hatching success does not change significantly after a 24h exposure to -2 °C in the F1 generation [106] and another study found the length of the egg maturation period does not greatly effect survival rates [96]. We thus make the assumption that only the absolute minimum temperature experienced throughout the winter is shaping the survival curve. This survival probability is then applied only when eggs are hatching in spring. This is in line with the findings of [107] whose fitted daily winter mortality was too high to give meaningful results. In the end, we used a curve fitted to survival data of Italian eggs against minimum temperatures [105] (Fig. S2).
There are some studies looking into the interaction of temperature and larval densities on mortality of *Aedes* mosquitoes but they do not show a clear pattern [108]. We thus keep temperature-related and density-related mortalities separated.

Diapause

The relationship between latitude, L, and the critical photo period $\mathrm{CPP_A}$ after which half of the eggs laid go into a diapausing state shows a positive correlation [109]. We use the observations made in Japan and in the US after adaptation to derive a linear function for our model, see Figure S3.

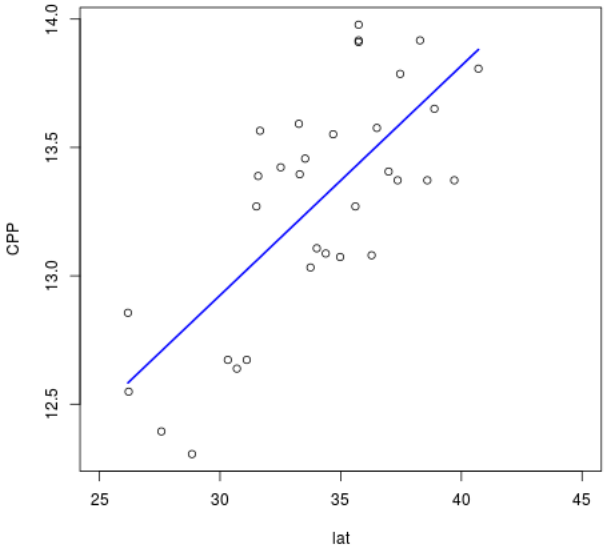

Figure S3: Relationship between latitude, L, and the critical photo period $\mathrm{CPP_A}$ in Japan and the US after adaptation [109].

The hatching of diapausing eggs in spring is triggered by a critical temperature, $\mathrm{CTT_S} = 11\,°\mathrm{C}$ and photo period, $\mathrm{CPP_S} = 11.25$ hours [110]. Eggs then only hatch if mean temperatures over a period of 7 days reach this threshold [111].

SI.2 Model with 7 stages

Other studies use larvae and pupae instead of juveniles to model immature development, some studies also use host-seeking and gestating stages to model the gonotrophic cycle [112, 113, 111]. An advantage of additional stages is that the development rate from egg to adult can approximate a Gamma distribution, which is more realistic with delayed development (linear chain trick) [114].
We tested whether such an extended model structure with additional parameters (Fig. S5) improved the fit to observed container index (CI) data but it gave very similar results. Comparing model output with spatial observations failed to predict occurrences in parts of East Europe and north of 45°N. We thus chose the model with only five equations, as there is also more literature data available to parametrise the reduced model.

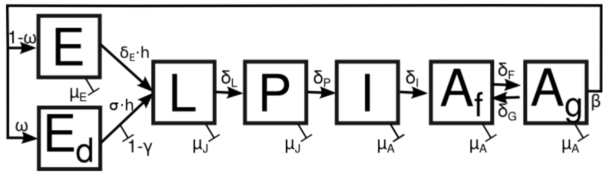

Figure S4: Model with juvenile class split up into a larva and a pupal class, and the mature female class split up into a host-seeking and a gestating/ovipositing class.

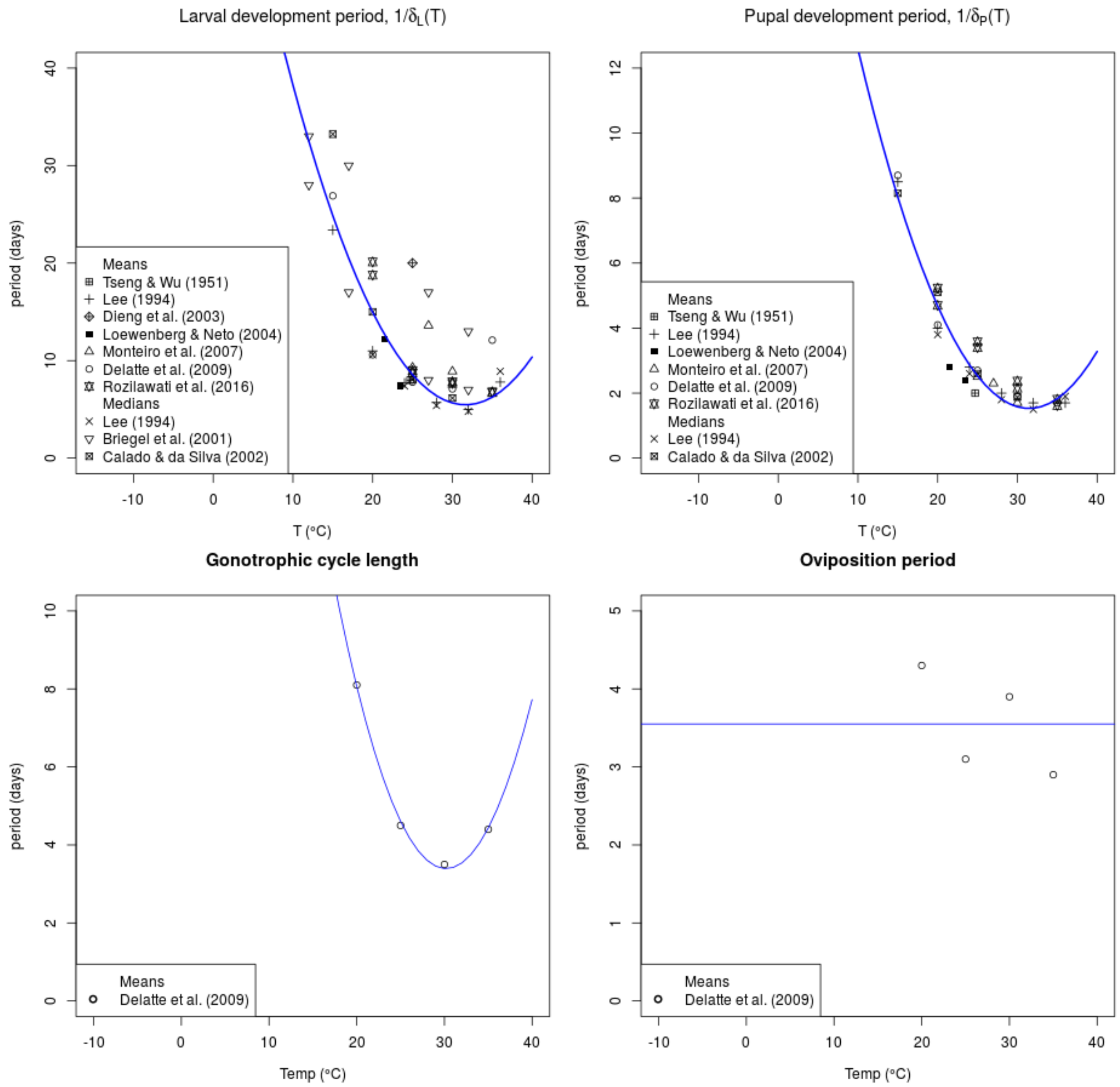

Figure S5: Development periods for larvae and pupae, as well as period from blood meal intake until oviposition (GTC) and period of oviposition. These are only used in the extended model with seven equations.

SI.3 Suitability index E_0

We define the suitability of a certain year and location as the number of diapausing eggs at the end of the year divided by the (small) number of diapausing eggs at the beginning of the year ($E_i = E_d(x = 365)/E_d(x = 1)$). In our model, diapausing eggs do not experience a daily mortality (a survival probability is applied in spring instead, see SI.1) and thus accumulate over autumn. At the end of December, when almost all adult females have died off and no more eggs are produced, diapausing egg numbers E_d have reached a stable plateau and $E_d(x = 365)$ can be used for E_0 calculation without taking an average over a certain period.

It would be also possible to use other stages for comparison, e.g. the number of adult females during the summer. It would be necessary though, to use an average over a certain time period for this variant, as numbers of juvenile or adults can fluctuate on a daily basis. As an example, we define

$$A_i = \frac{1/30 \sum_{x=201}^{230} A_i(x)}{1/30 \sum_{x=201}^{230} A_{i-1}(x)}$$

as the averaged number of females over 30 days in July/August of one year divided by the averaged number of females over the same period in the previous year. Figure S6 shows mosquito development when introducing diapausing eggs at DOY = 1 and running simulations for 2016 (left) to calculate E_0, and mosquito development when introducing adult females at DOY = 200 and running simulations for 2015 and 2016 (right) to calculate A_0.

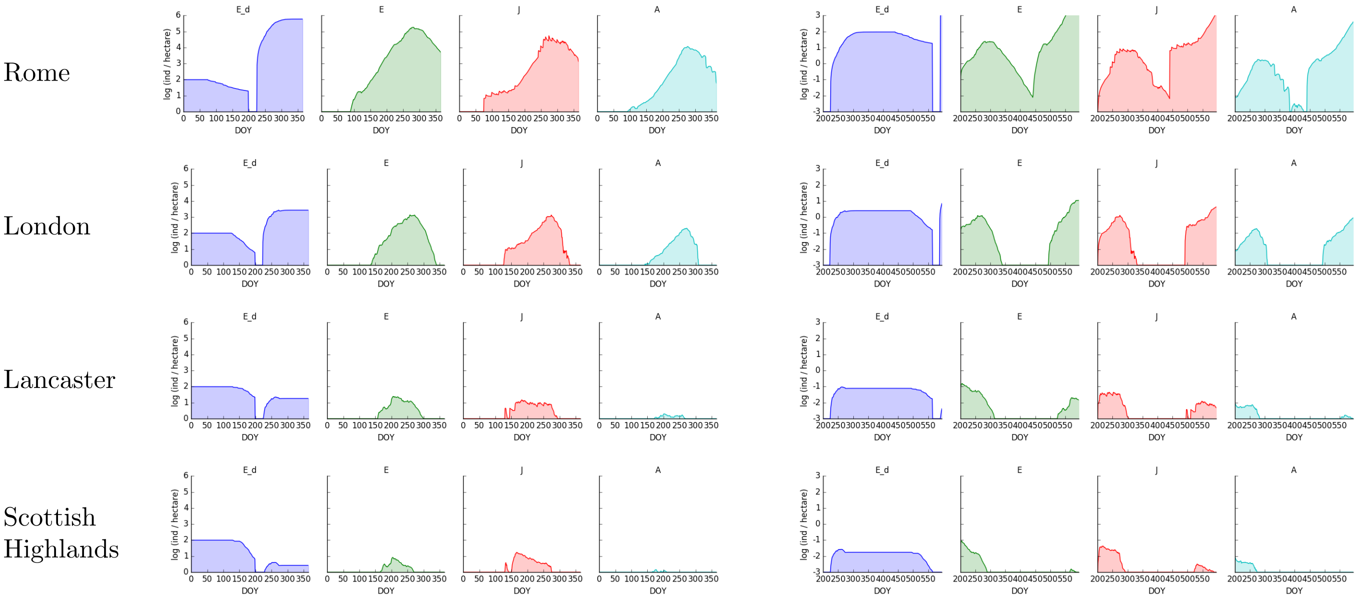

Figure S6: Mosquito population development Left plots show an introduction of $1 \cdot 10^2$ eggs per hectare at DOY = 1 for four locations, running the simulations for 365 days. E_i is then calculated by dividing the last time point of E_d by the first. Right plots show an introduction of $1 \cdot 10^{-2}$ females per hectare at DOY = 200 for the same locations, running the simulations for $2 \times 365 = 730$ days. A_i is then calculated by dividing the average of A for days 565 to 585 (days 200 to 230 of year 2), by the average of A for days 200 to 230 (of year 1). Please note the log-scale on the y-axis: When we remove diapausing eggs at DOY 200, we actually only remove a couple of eggs.

Resulting values for $E_0 = E_{2016}$ and $A_0 = A_{2016}$ are very similar, compare Table S1. Values for A_0 are slightly higher as relatively more females had been introduced than diapausing eggs.
The suitability definition based on diapausing eggs has the advantage that simulations only have to be run with single calendar years (often easier with climate data sets) and that population numbers do not have to

Table S1: Comparison of E_0 and A_0

**Location**	E_0	A_0	**Suitability**
Rome	6053.7	10243.8	Very suitable
London	27.3	32.7	Slightly suitable
Lancaster	0.18	0.23	Slightly unsuitable
Scottish Highlands	0.026	0.034	Very unsuitable

be averaged over long time periods and will thus use E_0 instead of A_0.

The definition of E_0 as the geometric mean of all E_i analysed has another advantage; $E_0^n = \Pi_{i=1}^n E_i$ gives an approximate value of population increase/decrease after n years; as an example let $E_1 = 3$, $E_2 = 0.5$ and $E_3 = 1.5$, then $E_0 = 1.3$ and thus slightly suitable, and $E_0^3 = 2.3$. Starting with one diapausing egg at the beginning of year 1, we can expect approximately 23 eggs at the end of year 3. This estimate does only hold in the first few years though, later on the mosquito population will approximate its carrying capacity and yearly egg numbers will more or less stagnate.

SI.4 Daylight model

Assuming sunrise/sunset is when the centre of the sun is even with the horizon, the daylight model [115] to calculate photoperiod, P, is given by

$$\theta = 0.2163108 + 2\,\tan^{-1}(0.9671396\,\tan(0.0086\,(\mathrm{DOY} - 186)))$$
$$\phi = \sin^{-1}(0.39795\,\cos(\theta))$$
$$P = 24 - \frac{24}{\pi}\cos^{-1}\left(\frac{\sin(\frac{\pi\,L}{180})\,\sin(\phi)}{\cos(\frac{\pi\,L}{180})\,\cos(\phi)}\right).$$

with DOY being day of year, ranging from 1 to 365, and L for latitude in degrees.
We approximate the time of sunrise, t_s, by

$$t_s = \frac{12}{\pi}\cos^{-1}\left(\frac{\sin(\frac{\pi L}{180})\,\sin(\phi)}{\cos(\frac{\pi L}{180})\,\cos(\phi)}\right).$$

SI.5 2060s

The NASA-NEX GDDP project provides calibrated general circulation model (GCM) outputs (daily rainfall and temperature) for future emission scenarios (RCPs) and for historical experiments. 21 GCMs are available (https://nex.nasa.gov/nex/projects/1356/) at a spatial resolution of 0.25° (approximately $25\,km \times 25\,km$). As the dynamical life cycle model for *Ae. albopictus* is computationally expensive to run, we took a subset of five GCMs out of 21, while keeping those models that represent the variability in the ensemble, i.e. GCMs that maximize uncertainty. For this, we first calculated land annual temperature indices for all GCMs and each emission scenario. We then kept the GCMs that were closest to the minimum, 25th percentile, median, 75th percentile and maximum temperature increase in the ensemble of the 21 GCMs (using the RMSE that minimized the distance to each categories, Tab. S2). Please see [116] for full details.

Table S2: General circulation model and RMSE to closest category (min, 25th, 50th, 75th, max temperature increase).

	**RCP4.5**	**RMSE**	**RCP8.5**	**RMSE**
Minimum	inmcm4	0.485	inmcm4	0.512
25th quantile	MRI-CGCM3	0.347	CESM1-BGC	0.360
Median	NorESM1-M	0.366	NorESM1-M	0.336
75th quantile	CanESM2	0.333	CanESM2	0.326
Maximum	MIROC-ESM-CHEM	0.630	MIROC-ESM-CHEM	0.567

SI.6 Europe's suitability for 2016

Figure S7 shows the suitability for 2016 only. The suitability over Western Europe (UK, France, Benelux, Germany) is higher than the 10-year mean, compare Figure 2 in the main text. Note that the used climate data set was incomplete for 2016, so that northern Italy and areas around the Black Sea appear grey.

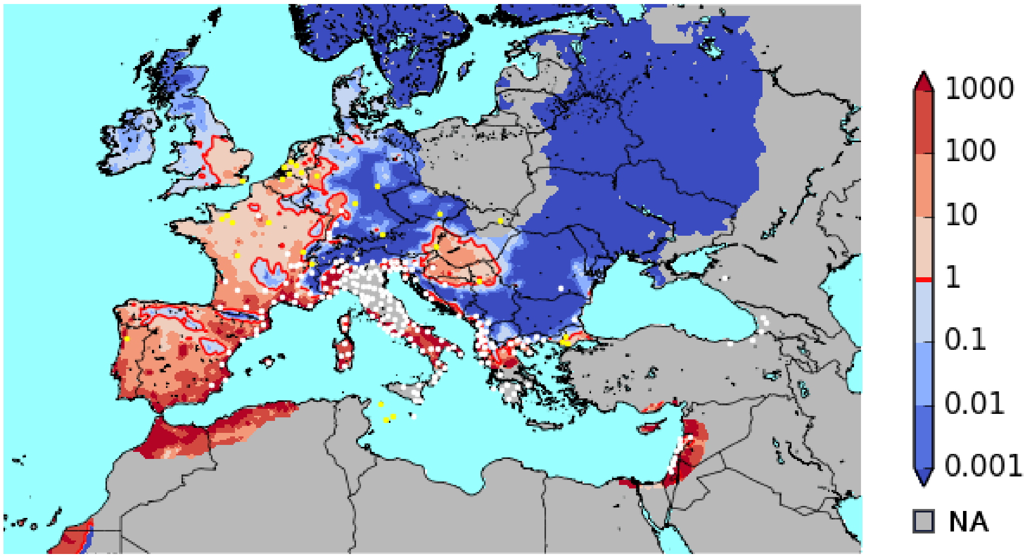

Figure S7: Suitability index for 2016 White dots show established *Ae. albopictus* populations, yellow dots show one-time sightings. Background colours show the simulated suitability index. Red contour distinguishes suitable ($E_0 > 1$) from unsuitable areas ($E_0 < 1$). The E-OBS climate data set has been used.

SI.7 Comparison with temporal container index data

Italian container index (CI) data of *Ae. albopictus* eggs are taken for a model validation in time. The data for 2011 – 2015 is available from http://www.zanzaratigreonline.it/ [117]. The region of Emilia-Romagna has nine districts and each district is monitored for *Ae. albopictus* eggs by ovitraps that are checked and numbers are counted on a biweekly basis. To exclude migration effects, we first checked if CI data was spatially correlated, which it was not, see Figure S8.

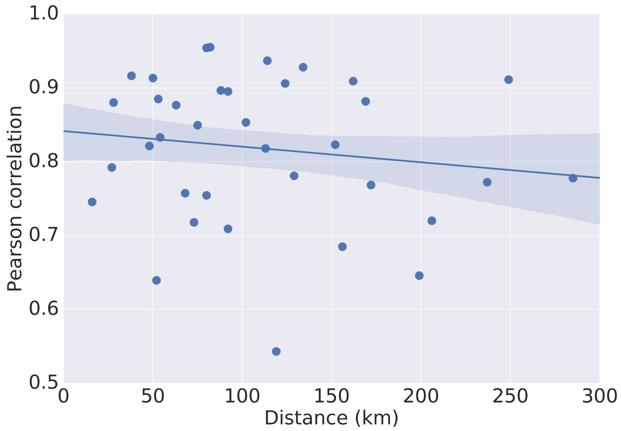

Figure S8: Test for spatial correlation Pearson's correlation coefficient between the nine regions' container index data plotted against distance between regions. Though the correlation between sampling sites is generally high, the slight negative trend between distance and Pearson correlation is not significant.

We run our model with Emilia-Romagna climate data for the period 2010 – 2015 to have one year of spin-up (cell codes 01421, 01867, 01699, 01138, 00369, 00774, 01983, 00977, 02231 for the according region). Figure S9 shows the comparison between the empirical CI data and scaled simulated egg data over a period of five years in the according region. Pearson's correlation coefficient for simulated vs. empirical egg data for individual regions range from $r = 0.54$ for Reggio-Emilia to $r = 0.86$ for Bologna, with an overall correlation of $r = 0.70$ (95% CI: $0.67 \leq r \leq 0.73$, N=996).

In addition, we look at the upper tercile (highest 33% of all biweekly values over all nine regions) and upper decile (highest 10%) of CI data and model outputs. The upper tercile indicates the main mosquito season while the upper decile indicates the highest peaks. Figure S10 shows that the model captures the main seasonality quite well, onset and end match approximately for observed and simulated numbers. However, the model overestimates the peak mosquito numbers in the years 2011 and 2014, while it underestimates peak mosquito numbers in 2015. This discrepancy is mainly due to 2014 showing very suitable spring temperatures and abundant rainfall in summer, while 2015 does not (not shown). True skill score TSS = 0.82 for upper terciles and TSS = 0.42 for upper deciles categories.

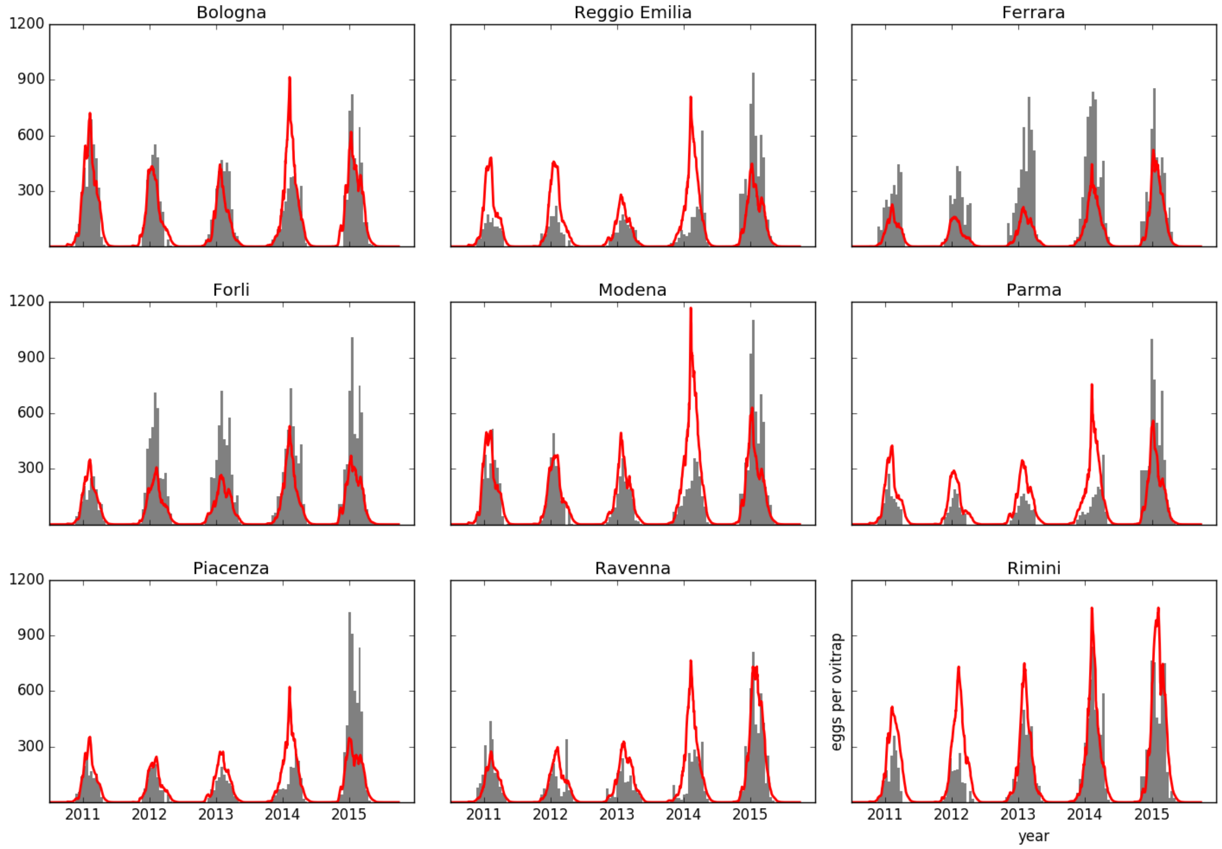

Figure S9: Temporal validation. Grey bars show observed *Ae. albopictus* container index data from nine regions in northern Italy. The red line indicates model output for laid mosquito eggs (divided by 6.3e4 for scale). The ERG5 Eraclito climate data set has been used to drive the model for 2010 – 2015 to give a one year spin-up.

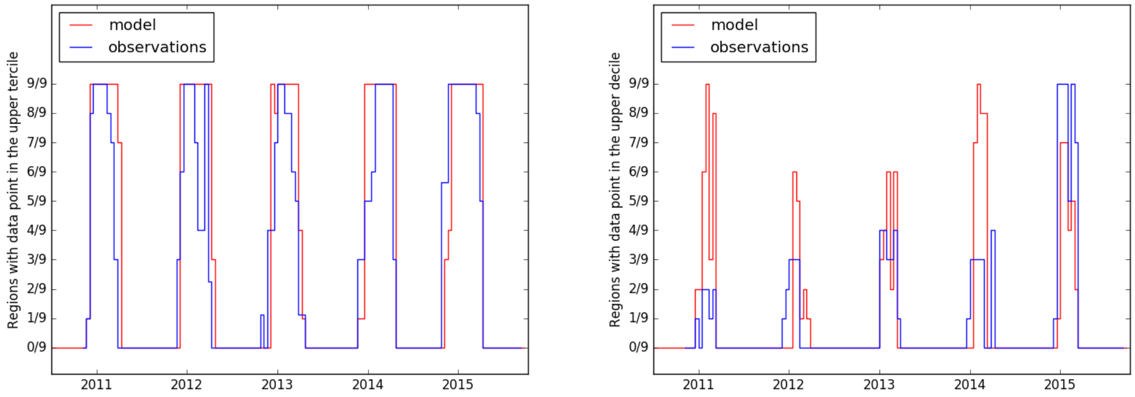

Figure S10: Comparing the upper tercile (left) and upper decile (right) between observed container index data and simulated egg data. If values of all nine regions lie in the upper tercile/decile of all data points for a specific date, the graph shows a value of 9/9.

SI.8 DTR vs. mean temperature runs

Running the model with and without DTR enables us to compare the suitability outputs. Figure S11 shows that the northern European regions (England, northern France, Germany, Czech Republic, Scandinavia) show an increased suitability in the model runs that consider the effect of DTR. Spain and the regions around Israel show decreased suitability, while the Balkan region is indifferent. The E-OBS rainfall data set is incomplete for northern Italy and north-east Africa in many years, so the high values in these regions might be not reliable.

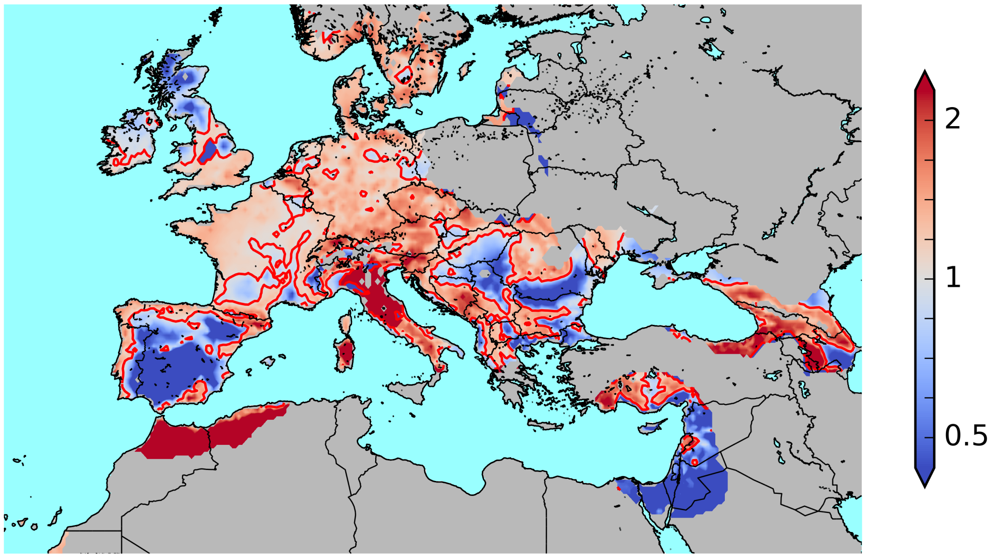

Figure S11: Suitability of the DTR run for 2006 – 2016, relative to the run using only daily mean temperatures. The red contour line indicates a value of 1, above which the DTR runs gives more suitable results, and below which the mean temperature runs do.

Including the DTR in simulations affects various parameters involved in population growth. One of the key parameters is the temperature-dependent development rate of juveniles, $\delta_J(T)$. This development rate is non-linear, with a maximum at around 30 °C, compare $1/\delta_J$ in Figure S1. Due to the non-linearity, being 8 °C above T_{mean} has a bigger positive effect on the mean development rate than being 8 °C below T_{mean}, if T_{mean} is relatively low. If T_{mean} is high, e.g. 30 °C, then any divergence from this optimal development temperature only decreases the rate. As a result, including a DTR in simulations can have positive or negative effects on population growth, depending on T_{mean}. Figure S12 shows the development of egg numbers over 365 days at constant and varying temperatures. For Figure 4 in the main text, we e.g. divide $E_{18\,^\circ C,DTR=8}(x = 365)$ by $E_{18\,^\circ C,DTR=0}(x = 365)$ to indicate relative population growth at $T_{\text{mean}} = 18\,^\circ\text{C}$ and DTR=8 °C. The fact that the population size has reached the carrying capacity at 28 °C after 365 days but not quite at 18 °C only has a small quantitative but not qualitative effect on the relative population growth.

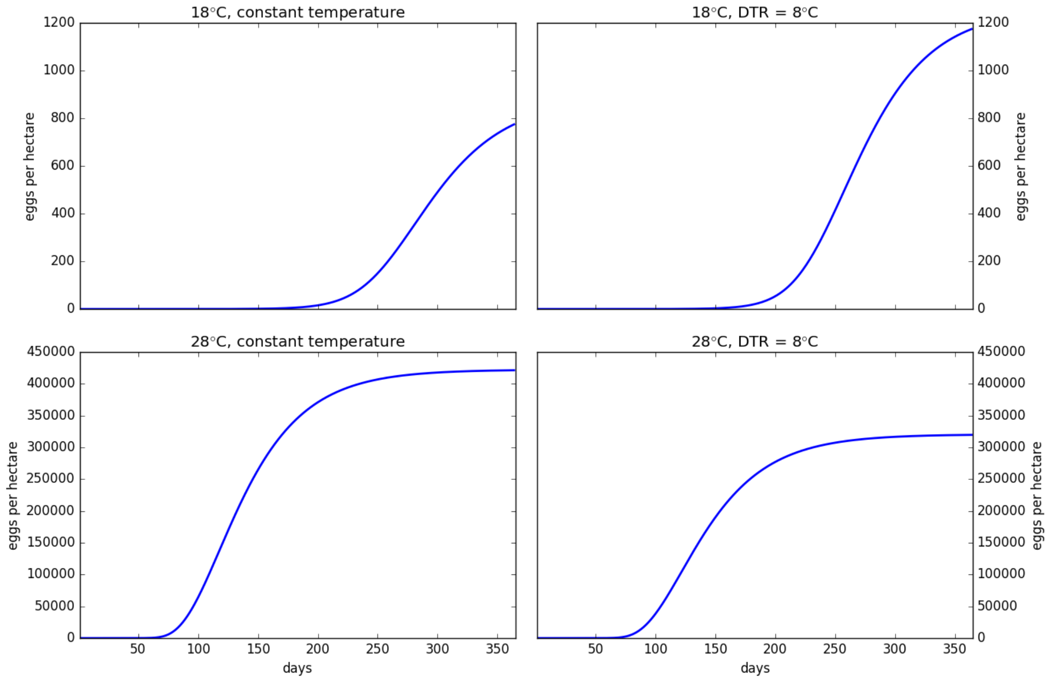

Figure S12: Influence of DTR over time Comparing the development of egg numbers over 365 days at constant 18 °C (top) and 28 °C (bottom) with and without a DTR of 8 °C. The DTR has a positive effect at low temperatures and a negative effect at higher temperatures.

SI.9 Equilibria & Stability

We analysed the system of differential equations for equilibria and their according stabilities. Assuming rainfall is not a limiting factor ($PR > 0$ and constant), we get a trivial equilibrium, $(E^*, J^*, I^*, A^*, E_d^*) = 0$, and a non-trivial equilibrium:

$$\begin{aligned}
A^* &= K\left(\left(h\,\delta_E\,\frac{\beta\,(1-\omega)}{\mu_e + h\,\delta_E} + \gamma\,\beta\,\omega\right)B - \delta_J - \mu_J\right)B \\
E^* &= \frac{\beta\,(1-\omega)}{\mu_E + h\,\delta_E}A^* \\
J^* &= \frac{1}{B}A^* \\
I^* &= \frac{\mu_A}{\delta_I}A^* \\
E_d^* &= \frac{\beta\,\omega}{h\,\sigma}A^*
\end{aligned}$$

with $B = \frac{\delta_J\,\delta_I}{2\,\mu_A\,(\mu_A + \delta_I)}$.

Checking the eigenvalues of the Jacobian shows that the trivial equilibrium is unstable between 13 °C and 32 °C, meaning that we have a positive equilibrium in this temperature range for any positive carrying capacity K. K is non-zero if rainfall or the human population density are non-zero. The optimum temperature that maximises population numbers is at 27 °C, close to what Brady et al. [98] have found for a maximum adult life span.

References

[72] Quinn BK. A critical review of the use and performance of different function types for modeling temperature-dependent development of arthropod larvae. Journal of Thermal Biology. 2017;63(November 2016):65–77. doi:10.1016/j.jtherbio.2016.11.013.

[73] Tseng S, Wu I. An ecological study of mosquitoes in Wuhan area. Bull Entomol Res. 1951;(42):527–533.

[74] Udaka M. Some ecological notes on Aedes albopictus Skuse in Shikoku, Japan. Japanese journal of entomology. 1959;27(3):202–208.

[75] Lee S. Development of eggs, larvae and pupae of Aedes albopictus (Skuse) (Diptera: Culicidae). Chinese Journal of Entomology. 1994;14(1):13–32.

[76] Löwenberg-Neto P, Navarro-Silva Ma. SYSTEMATICS , MORPHOLOGY AND PHYSIOLOGY Development , Longevity , Gonotrophic Cycle and Oviposition of Aedes albopictus Skuse (Diptera: Culicidae) under Cyclic Temperatures. Neotropical Entomology. 2004;33(February):29–33. doi:10.1590S1519-566X2004000100006.

[77] Delatte H, Gimonneau G, Triboire A, Fontenille D. Influence of Temperature on Immature Development, Survival, Longevity, Fecundity, and Gonotrophic Cycles of Aedes albopictus , Vector of Chikungunya and Dengue in the Indian Ocean. Journal of Medical Entomology. 2009;46(1):33–41. doi:10.1603/033.046.0105.

[78] Calado DC, Navarro-Silva MA. Influência da temperatura sobre a longevidade, fecundidade e atividade hematofágica de Aedes (Stegomyia) albopictus Skuse, 1894 (Diptera, Culicidae) sob condições de laboratório. Revista Brasileira de Entomologia. 2002;46(1):93–98. doi:10.1590/S0085-56262002000100011.

[79] Tsuda Y, Tagaki M, Suzuki A, Wada Y. A Comparative Study on Life Table Characteristics of Two Strains of Aedes albopictus from Japan and Thailand. Tropical medicine. 1994;36(1):15–20.

[80] Dieng H, Boots M, Mwandawiro C, Satho T, Hasegawa M, Nyambura GJ, et al. Effects of a copepod predator on the survivorship and development of Aedes albopictus (Diptera: Culicidae). Med Entomol Zool. 2003;54(2):187–192.

[81] Dickerson CZ. The effects of temperature and humidity on the eggs of Aedes aegypti (L.) and Aedes albopictus (Skuse) in Texas; 2007.

[82] Monteiro LCC, de Souza JRB, de Albuquerque CMR, et al M. Eclosion rate, development and survivorship of Aedes albopictus (Skuse)(Diptera: Culicidae) under different water temperatures. Neotropical entomology. 2007;36(6):966–971. doi:10.1590/S1519-566X2007000600021.

[83] O'Donnell DL. Ecology and Population Differentiation of the Asian Tiger Mosquito, Aedes Albopictus; 2009.

[84] Hashim NA, Dieng H, Satho T, Nurita AT, Salmah MC, Miake F, et al. The biology and demographic parameters of Aedes albopictus in northern peninsular Malaysia. Asian Pacific Journal of Tropical Biomedicine. 2011;1(6):472–477. doi:10.1016/S2221-1691(11)60103-2.

[85] Farjana T, Tuno N, Higa Y. Effects of temperature and diet on development and interspecies competition in Aedes aegypti and Aedes albopictus. Medical and Veterinary Entomology. 2011;26(2):1–8. doi:10.1111/j.1365-2915.2011.00971.x.

[86] Rozilawati H, Masri SM, Tanaselvi K, Zairi J, Nazn W, Lee H. Effect of temperature on the immature development of Aedes albopictus Skuse. Southeast Asian Journal of Tropical Medicine and Public Health. 2016;47(4):731–746.

[87] Briegel H, Timmermann SE. Aedes albopictus (Diptera : Culicidae): Physiological Aspects of Development and Reproduction. Journal of Medical Entomology. 2001;38(4):566–571.

[88] Calado DC, Navarro da Silva MA. Evaluation of the temperature influence on the development of Aedes albopictus. Revista de Saude Publica. 2002;36(2):173–179.

[89] Westbrook CJ, Reiskind MH, Pesko KN, Greene KE, Lounibos LP, et al W. Larval Environmental Temperature and the Susceptibility of Aedes albopictus Skuse (Diptera: Culicidae) to Chikungunya Virus. Vector borne and zoonotic diseases. 2010;10(3):241–247. doi:10.1089/vbz.2009.0035.

[90] Gubler DJ, Bhattacharya NC. Observations on the reproductive history of Aedes (Stegomyia) albopictus in the laboratory; 1971.

[91] Hashim NA, Ahmad AH, Rawi CSM, Tahir NA, Basari N. Life tables study of immature aedes albopictus (Skuse) (Diptera: Culicidae) during the wet and dry seasons in Penang, Malaysia. Southeast Asian Journal of Tropical Medicine and Public Health. 2008;39(1):39–47.

[92] Braks M, Juliano S, Lounibos LP. Superior reproductive success on human blood without sugar is not limited to highly anthropophilic mosquito species. Med Vet Entomol. 2006;20(1):53–59. doi:10.1097/MCA.0000000000000178.Endothelial.

[93] Islam MS, Dobson SL. Wolbachia effects on Aedes albopictus (Diptera: Culicidae) immature survivorship and development. Journal of medical entomology. 2006;43(4):689–695. doi:10.1603/0022-2585(2006)43[689:WEOAAD]2.0.CO;2.

[94] Kamimura K, Matsuse IT, Takahashi H, Komukai J, Fukuda T, Suzuki K, et al. Effect of temperature on the development of Aedes aegypti and Aedes albopictus. 2002;53(1):53–58.

[95] Chang Lh, Hsu El, Teng Hj, Ho CM. Differential Survival of Aedes aegypti and Aedes albopictus (Diptera : Culicidae) Larvae Exposed to Low Temperatures in Taiwan. Journal medical entomology. 2007;44(2):205–210.

[96] Gubler DJ. Comparison of reproductive potentials of Aedes (Stegomyia) albopictus Skuse and Aedes (Stegomyia) polynesiensis Marks. Mosquito News. 1970;30(2):201–209.

[97] Juliano SA, O'Meara GF, Morrill JR, Cutwa MM, Meara GFO, Cutwa MM. Desiccation and thermal tolerance of eggs and the coexistence of competing mosquitoes. Oecologia. 2002;130(3):458–469. doi:10.1007/s004420100811.

[98] Brady, Johansson, Guerra, Bhatt, Golding, Pigott, et al. Modelling adult Aedes aegypti and Aedes albopictus survival at different temperatures in laboratory and field settings. Parasites & vectors. 2013;6:351. doi:10.1186/1756-3305-6-351.

[99] Bonnet DD, Worcester DJ. The dispersal of Aedes albopictus in the territory of Hawaii. The American journal of tropical medicine and hygiene. 1946;26:465–476.

[100] Takagi M, Tsuda Y, Suzuki A, Wada Y. Movement of individually marked Aedes albopictus females in Nagasaki, Japan. Tropical Medicine. 1995;37(2):79–85.

[101] Maciel-de Freitas R, Neto RRB, Gonçalves JMJ, Codeço CCT, Lourenço-de Oliveira R. Movement of dengue vectors between the human modified environment and an urban forest in Rio de Janeiro. Journal of Medical Entomology. 2006;43(6):1112–1120. doi:http://dx.doi.org/10.1603/0022-2585(2006)43[1112:MODVBT]2.0.CO;2.

[102] Lacroix AR, Delatte H, Hue T, Reiter P, Lacroix R. Dispersal and Survival of Male and Female Aedes albopictus (Diptera: Culicidae) on Réunion Island. America. 2009;46(5):1117–1124.

[103] Marini F, Caputo B, Pombi M, Tarsitani G, Della Torre A. Study of Aedes albopictus dispersal in Rome, Italy, using sticky traps in mark-release-recapture experiments. Medical and Veterinary Entomology. 2010;24(4):361–368. doi:10.1111/j.1365-2915.2010.00898.x.

[104] Hawley W, Reiter P, Copeland R, Pumpuni C, Craig G. Aedes albopictus in North America: probable introduction in used tires from northern Asia. Science. 1987;236(4805):1114–1116. doi:10.1126/science.3576225.

[105] Thomas S, Obermayr U, Fischer D, Kreyling J, Beierkuhnlein C. Low-temperature threshold for egg survival of a post-diapause and non-diapause European aedine strain, Aedes albopictus (Diptera: Culicidae). Parasites & Vectors. 2012;5(1):100. doi:10.1186/1756-3305-5-100.

[106] Kreß A, Oppold AM, Kuch U, Oehlmann J, Müller R. Cold tolerance of the Asian tiger mosquito Aedes albopictus and its response to epigenetic alterations. Journal of Insect Physiology. 2017;99(April):113–121. doi:10.1016/j.jinsphys.2017.04.003.

[107] Erguler K, Smith-Unna SE, Waldock, Proestos, Christophides, Lelieveld, et al. Large-scale modelling of the environmentally-driven population dynamics of temperate aedes albopictus (Skuse). PLoS ONE. 2016;11(2):1–28. doi:10.1371/journal.pone.0149282.

[108] Hapairai L, Marie J, Sinkins S, Bossin H. Effect of temperature and larval density on Aedes polynesiensis (Diptera: Culicidae) laboratory rearing productivity and male characteristics. Acta Trop. 2014;p. 108–115. doi:10.1016/j.actatropica.2013.11.024.

[109] Urbanski J, Mogi M, O'Donnell D, DeCotiis M, Toma T, Armbruster P. Rapid Adaptive Evolution of Photoperiodic Response during Invasion and Range Expansion across a Climatic Gradient. American Naturalist. 2012;179(4):490–500. doi:10.1086/664709.

[110] Toma L, Severini F, Di Luca M, Bella A, Romi R. Seasonal patterns of oviposition and egg hatching rate of Aedes albopictus in Rome. Journal of the American Mosquito Control Association. 2003;19(1):19–22.

[111] Jia P, Lu L, Chen X, Chen J, Guo L, Yu X, et al. A climate-driven mechanistic population model of Aedes albopictus with diapause. Parasites & vectors. 2016;9(1):175. doi:10.1186/s13071-016-1448-y.

[112] Erickson RA, Presley SM, Allen LJS, Long KR, Cox SB. A stage-structured, Aedes albopictus population model. Ecological Modelling. 2010;221(9):1273–1282. doi:10.1016/j.ecolmodel.2010.01.018.

[113] Tran A, L'Ambert G, Lacour G, Benoit R, Demarchi M, Cros M, et al. A rainfall- and temperature-driven abundance model for Aedes albopictus populations. International Journal of Environmental Research and Public Health. 2013;10(5):1698–1719. doi:10.3390/ijerph10051698.

[114] Smith H. An Introduction to Delay Differential Equations with Applications to the Life Sciences. vol. 57; 2011. doi:10.1007/978-1-4419-7646-8.

[115] Forsythe WC, Rykiel EJ, Stahl RS, Wu Hi, Schoolfield RM. A model comparison for daylength as a function of latitude and day of year. Ecological Modelling. 1995;80(1):87–95. doi:10.1016/0304-3800(94)00034-F.

[116] Jones A, Turner J, Caminade C, Heath A, Wardeh M, Kluiters G, et al. Bluetongue risk under future climates. Nature Climate Change. 2019;p. 153–157. doi:10.1038/s41558-018-0376-6.

[117] Carrieri M, Albieri A, Angelini P, Baldacchini F, Venturelli C, Zeo SM, et al. Surveillance of the chikungunya vector Aedes albopictus (Skuse) in Emilia-Romagna (northern Italy): Organizational and technical aspects of a large scale monitoring system. Journal of Vector Ecology. 2011;36(1):108–116. doi:10.1111/j.1948-7134.2011.00147.x.
